# A Case Report of Splenic Rupture Secondary to Underlying Angiosarcoma

**DOI:** 10.7759/cureus.9439

**Published:** 2020-07-28

**Authors:** Brooke E Kania, Sugam Vasani

**Affiliations:** 1 Internal Medicine, United Hospital Center, Bridgeport, USA; 2 General Surgery, United Hospital Center, Bridgeport, USA

**Keywords:** splenic angiosarcoma, splenic rupture, splenectomy, gastrointestinal hemorrhage, neoplasm

## Abstract

Primary splenic angiosarcoma is a rare type of cancer that has not been well-illustrated due to infrequency and variability in patient presentation. Limited systemic therapy regimens for splenic angiosarcoma make early detection preferable, as management focuses on monitoring for recurrence and metastatic spread or preventing hemorrhagic complications of tumor burden such as splenic rupture. This cancer, in particular, is aggressive, and metastasis is common. Here, we discuss a 68-year-old female who presented with a splenic laceration caused by an underlying primary splenic angiosarcoma. The purpose of this article is to describe a patient who presents with noteworthy clinical features and a rare complication of splenic angiosarcoma to aid in the treatment and diagnosis of future patients.

## Introduction

Splenic angiosarcoma represents a rare malignancy that is aggressive in nature, primarily derived from splenic endothelial cells [1-2]. Patients diagnosed with this malignancy are most commonly over the age of 40 years; however, additional risk factors specific to angiosarcoma of the spleen have yet to be characterized [1-2]. The clinical presentation of splenic angiosarcoma is varied and nonspecific, with the most frequent clinical signs and symptoms being weakness, unintentional weight loss, dyspnea, and back pain [1-3]. The pathophysiology of this disease has not been sufficiently researched, as angiosarcoma can arise and manifest differently depending on the primary organ and particular endothelial cells involved [4]. A variety of systemic therapeutic regimens are being explored through clinical trials. Prophylactic splenectomy prior to splenic rupture has shown to be beneficial for long-term survival; however, the prognosis, in general, is unfavorable [3].

## Case presentation

A 68-year-old female presented to the emergency department (ED) via emergency medical services (EMS), with a chief complaint of worsening left flank pain radiating to the umbilicus for the past two days. Prior to the ED arrival, she had a syncopal episode where she fell on her left side. The review of systems was noteworthy for subjective fever, chest pain, dyspnea, headache, nausea, vomiting, and diarrhea. Past medical history was significant for arteriovenous malformation of the colon without hemorrhage, deep venous thrombosis, fibromyalgia, Helicobacter pylori infection, medullary sponge kidney, and bipolar disorder. Past surgical history was significant for surgical hernia repair, salpingo-oophorectomy, and tonsillectomy. The patient’s most recent dose of Xarelto 20 mg was taken the morning of the presentation. Family history was notable for cancer in multiple immediate family members. The patient was a never-smoker and denied alcohol use. She was a retired professional, and her medical power of attorney was present during the encounter.

**Figure 1:**
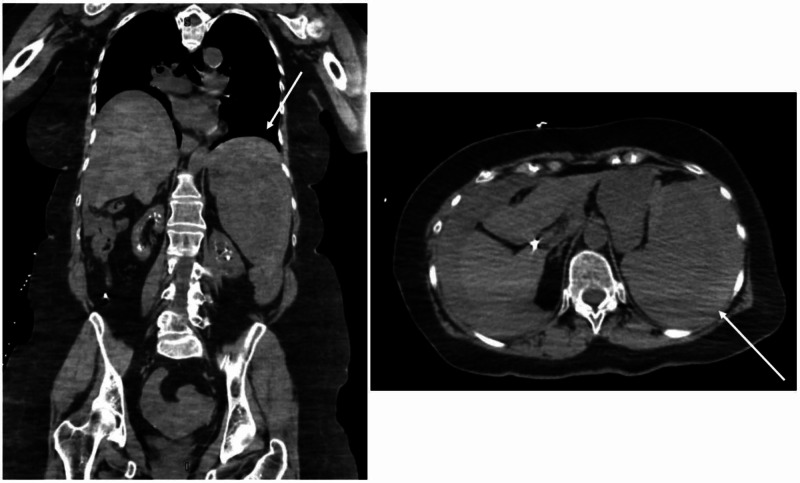
Initial CT chest, abdomen, and pelvis w/o IV contrast Significant for dense, abnormal fluid within the abdomen and pelvis Right) Coronal view; Left) Axial view IV: intravenous

During evaluation in the ED, the patient was hypotensive with a blood pressure of 59/42 millimeters of mercury (mmHg). She was tachycardic, with a heart rate of 105 beats per minute (bpm) and tachypneic with a respiratory rate of 23 breaths per minute. The physical exam was notable for an ill-appearing female in moderate distress. The abdominal exam was significant for diffuse tenderness with the absence of rebound or guarding. Labs were significant for leukocytosis, with a white blood cell count (WBC) of 19.7 x 10^3/μL, anemia with hemoglobin (Hb), and hematocrit (Hct) levels of 10.9 g/dL and 33.2%, respectively, and severe thrombocytopenia with a platelet level of 73 x 10^3/μL. The international normalized ratio (INR) was elevated at 2.84. The chemistry was pertinent for elevated blood urea nitrogen (BUN) of 24 mg/dL and creatinine elevation of 2.00 mg/dL, as well as hyperglycemia with a glucose level of 339 mg/dL. Lactic acidosis with a lactate level of 6.9 mmol/L was significant at the initial presentation. Baseline labs during office visits prior to ED presentation were within normal limits. Due to hemodynamic instability, platelet transfusion was initiated as well as 4 units of packed red blood cells. On evaluation for syncope, the electrocardiogram was notable for sinus tachycardia. Focused Assessment with Sonography for Trauma (FAST) Exam was concerning for hemoperitoneum. Once the patient was hemodynamically stable, additional radiology images were performed. Computed tomography (CT) chest showed clear lung fields with the absence of pneumothorax. CT abdomen and pelvis displayed a significant amount of dense, abnormal fluid within the abdomen and pelvis (Figure 1). Radiology reported an abnormal spleen with a large subcapsular hematoma and very likely splenic laceration. Chronic changes within the kidneys were noted, consistent with the patient’s diagnosis of medullary sponge kidney. No evidence of free air or obstruction was noted on CT. CT brain was unremarkable. All imaging was done without intravenous (IV) contrast due to chronic kidney disease, which limited the visualization of the patient’s injury.

She was admitted to the hospital inpatient floor for hemorrhagic shock secondary to solid organ injury and hemoperitoneum on Xarelto. Surgery was consulted and deemed this case a surgical emergency. The patient was taken directly to the operating room for an exploratory laparotomy, which confirmed capsular degloving of the spleen with a large hemoperitoneum. A splenectomy was performed with no complications. The patient was stabilized and was discharged 12 days following surgery. Ultimately, the patient returned to the emergency department one day following discharge due to two episodes of hematochezia. She described the blood as bright red and denied chest pain, dyspnea, syncope, nausea, vomiting, hemoptysis, dysuria, or rectal pain. She had not resumed her Xarelto since splenic rupture and no decrease in hemoglobin was noted. The one episode of bleeding quickly resolved upon arrival, and it was suspected to be due to hemorrhoids. The patient was discharged the following day with close follow-up.

**Figure 2:**
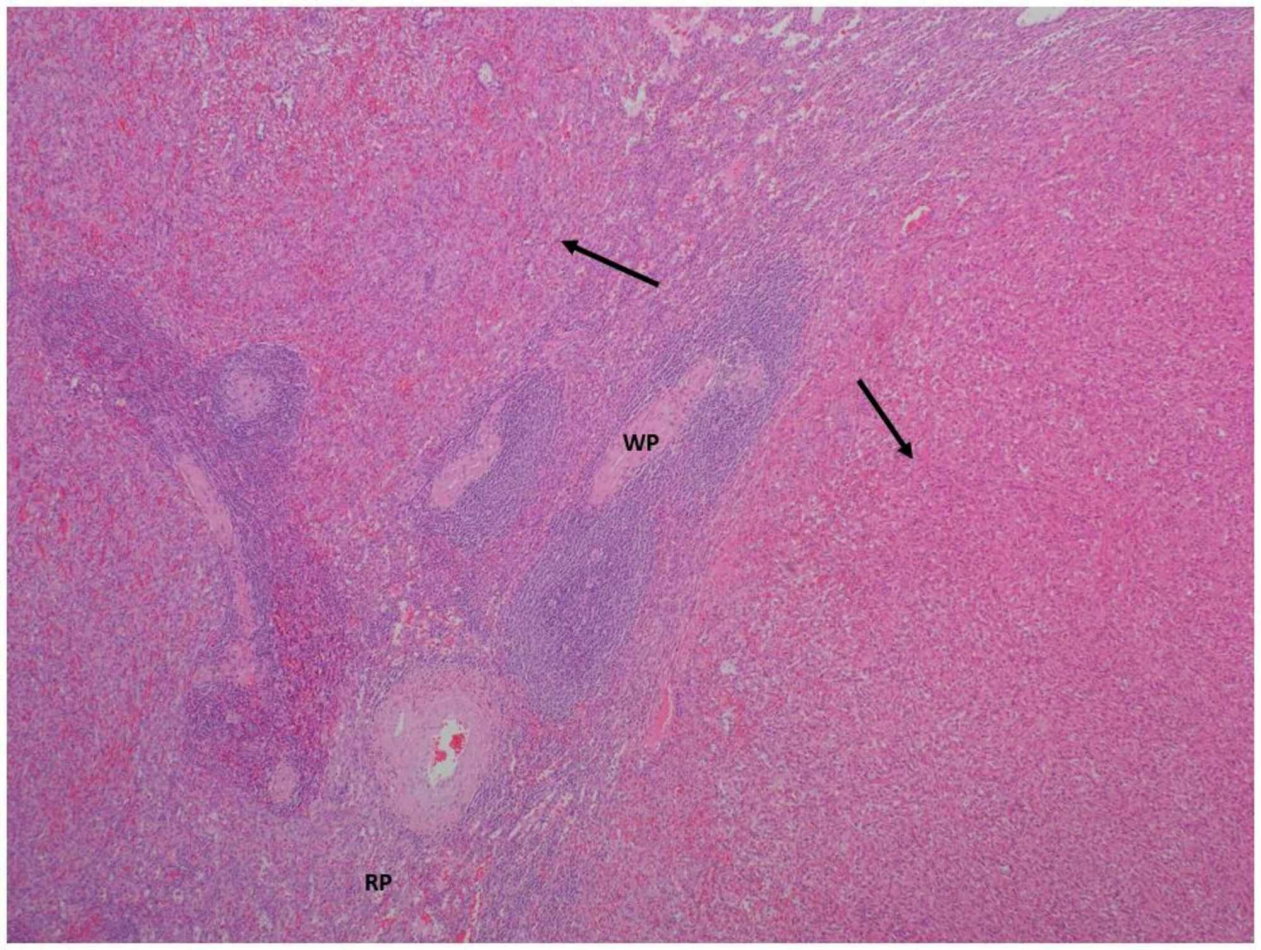
Hematoxylin and eosin-stained image of the spleen, low power Normal splenic white pulp (WP) and red pulp (RP) adjacent to the invading nodules of spindle cells forming thin, narrow vascular channels encompassing erythrocytes (arrows)

**Figure 3:**
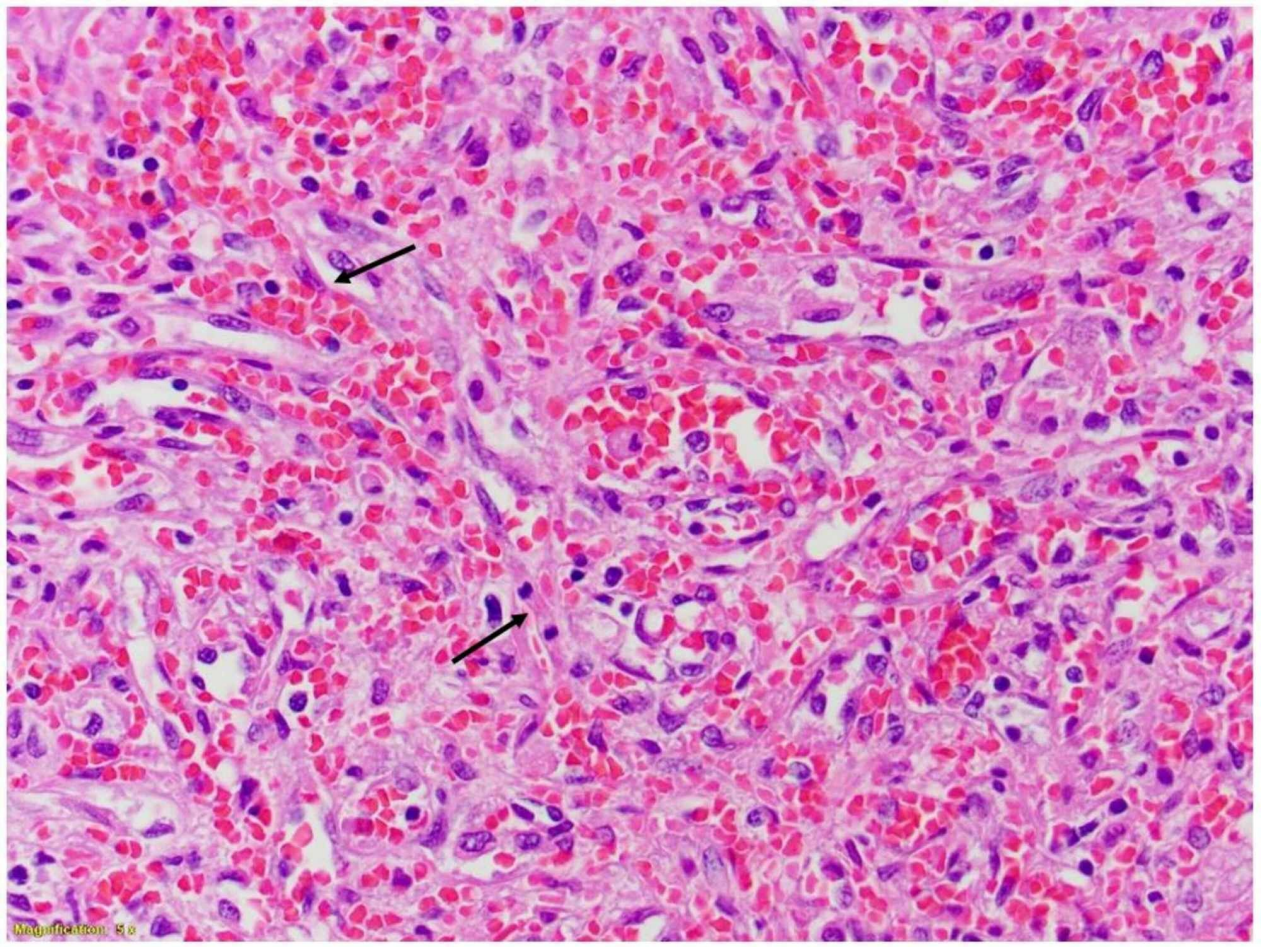
Hematoxylin and eosin-stained image of the spleen Neoplastic endothelial nuclei with mitoses forming anastomosing vascular channels

**Figure 4:**
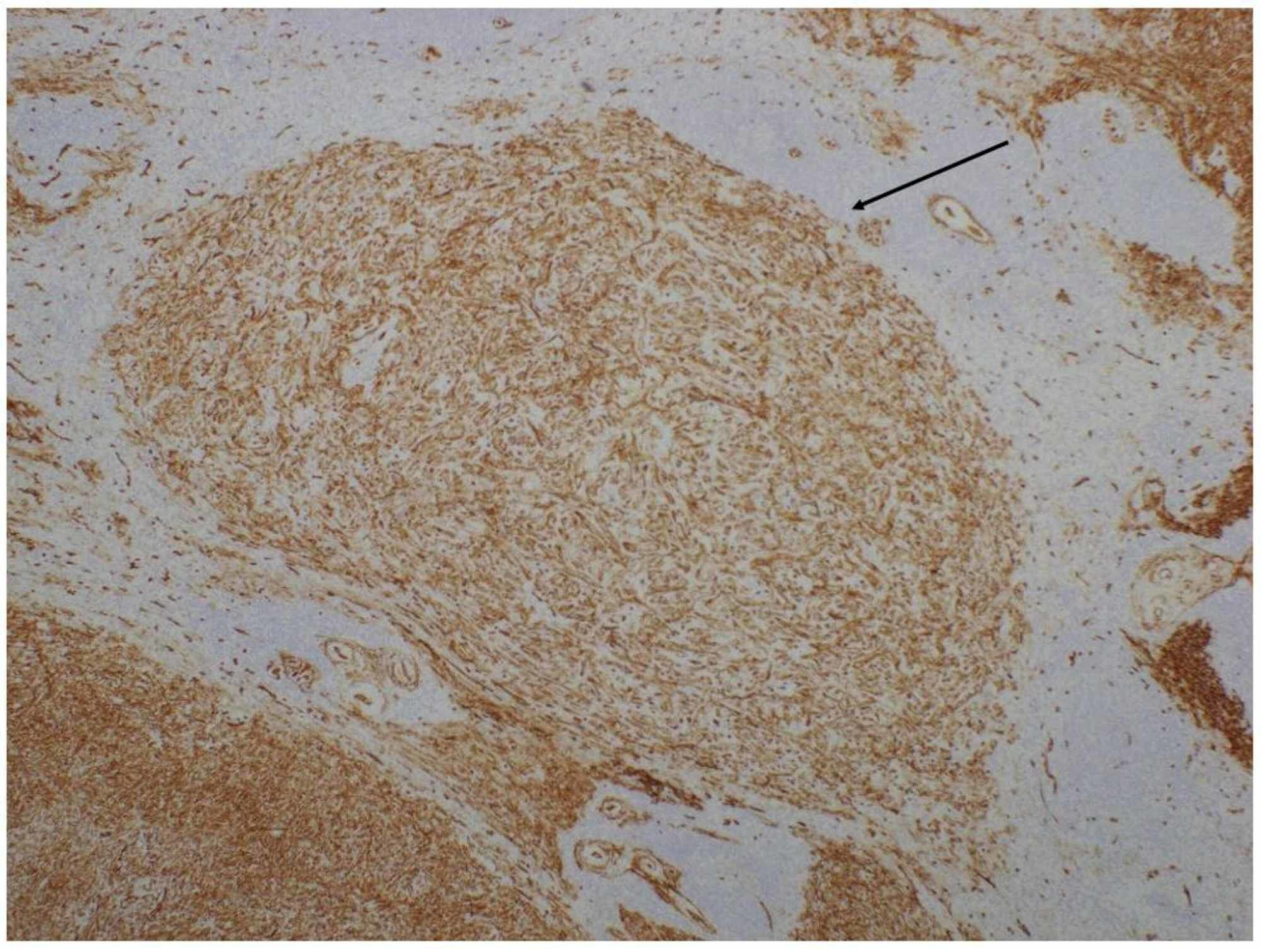
Specimen of the spleen, CD34 immunostain CD34 immunostain positive nodules containing spindle cells, consistent with an endothelial origin

Immunohistochemistry from the patient’s splenectomy specimen was notable for malignant angiosarcoma encompassing the organ entirely, with tumor measurements of 15.8 x 10.5 x 6.1 cm (Figures 2-4). The patient was referred to oncology where she had a CT chest, abdomen, and pelvis with IV contrast that suggested no evidence of metastatic disease. IV contrast imaging was warranted considering suspicion for cancer and improving acute kidney injury. Imaging revealed new bilateral pleural effusions most likely secondary to recent splenectomy procedure. Brain MRI with and without contrast was negative for metastases. Due to the infrequency of the patient’s diagnosis, she was referred for a second opinion to the University of Pittsburgh Medical Center (UPMC) and the decision was made to monitor the patient for recurrence.

**Figure 5:**
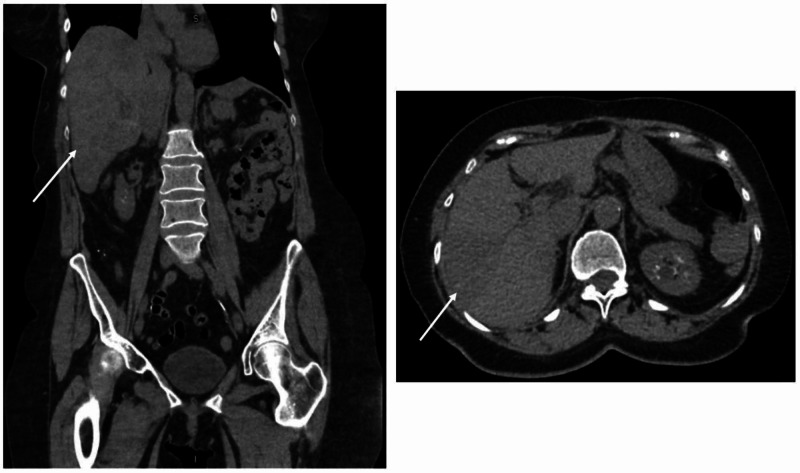
CT abdomen, pelvis: re-evaluation for metastatic disease Significant for hepatomegaly, with multiple areas of hypodensity concerning for metastasis Right) Coronal view; Left) Axial view CT: computed tomography

**Figure 6:**
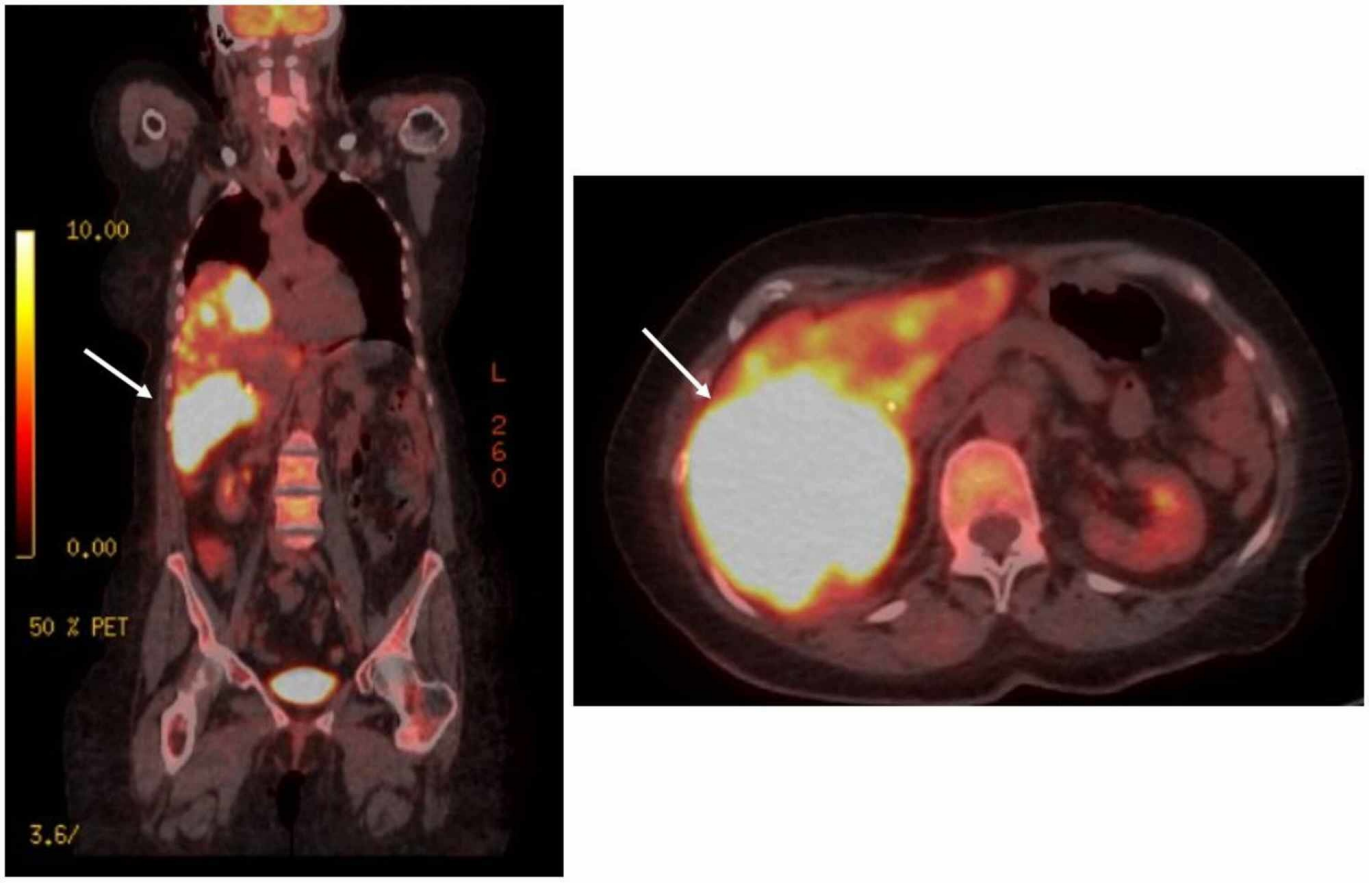
PET-CT: re-evaluation for metastatic disease Confirmation of metastatic disease, extensive liver involvement with multiple large hyperbolic hepatic metastases Right) Coronal view; Left) Axial view PET-CT: positron emission tomography-computed tomography

Within three months, the patient returned to the ED for right upper quadrant (RUQ) pain. Physical exam was unremarkable, with exception to minimal RUQ tenderness to palpation. She was found to have elevated liver enzymes, with aspartate transaminase (AST) of 122 U/L, alanine transaminase (ALT) of 102 U/L, and alkaline phosphatase (ALP) of 524 U/L. Repeated CT abdomen pelvis discovered hepatomegaly with multiple hypodense areas concerning for metastatic disease (Figure 5). Positron emission tomography-computed tomography (PET-CT) confirmed the presence of extensive liver involvement with multiple large hypermetabolic hepatic metastases (Figure 6). The patient was deemed a poor candidate for systemic chemotherapy or radiation therapy and the prognosis was dismal due to stage IV cancer. With her family’s support, the patient decided to proceed with hospice.

## Discussion

The incidence of splenic angiosarcoma represents a 1:1 male to female ratio, with a mean age of 40-55 years [1,4]. Our patient’s clinical presentation coincides well with the common chief complaints of patients with this rare, aggressive disease. Some risk factors identified with angiosarcoma, in general, include radiation exposure, chronic lymphedema, exposure to exogenous toxins, such as thorium dioxide, vinyl chloride, arsenic, and inherited disorders [5-6]. However, these risk factors have not been extensively studied with primary splenic angiosarcoma and, therefore, there are no clear risk factors for this specific disease [2,5]. As this cancer is derived from the lining of blood vessels, the spread is hematogenous and splenic rupture can worsen prognosis due to dissemination [3,7].

The pathophysiology of splenic angiosarcoma is variable due to the different tumors arising from the soft tissue and differences in histologic findings [4]. On microscopic evaluation, tumors are often described as heterogenous, with formations of vessels lined with atypical endothelial cells [2]. In terms of immunohistochemistry staining, tumors can be positive for various markers of vascular development such as factor VIII related antigen (FVIIIRAg), vascular endothelial growth factor receptor 3 (VEGFR3), cluster of differentiation 31 (CD31), and CD34 [2]. Additionally, markers displaying histiocytic development can also stain positive such as CD68 or lysozyme [2]. Our patient’s splenic pathology correlates with these findings, as her spleen had atypical spindle cells with positive CD34 immunostaining, confirming endothelial origin. Mitotic counts and tumor burden represent prognostic factors for splenic angiosarcoma [8]. Our patient’s tumor weighed 439 grams, encompassing the entirety of her spleen, and the mitotic rate was 6 per 10 high power fields (18 mitoses/mm^2^). With our patient and former studies, splenic angiosarcomas are thought to derive from splenic lining cells, although additional studies are needed to confirm this association [2].

Although clinical features vary and are not well-characterized, patients most commonly report left upper quadrant abdominal pain [1-3]. Additional symptoms have included generalized weakness, unexplained weight loss, dyspnea, and back pain [3]. Our patient initially presented with gastrointestinal complaints as well as flank pain radiating to the umbilicus. On physical exam, patients with splenic angiosarcoma may exhibit splenomegaly or an abdominal mass [1-3]. Labs may be significant for normochromic, normocytic anemia [3]. A rare complication of this disease is splenic rupture, which our patient ultimately experienced [1,3].

Abdominal CT may display splenic differentiation such as splenomegaly with contrast-induced heterogeneous enhancement and areas of necrosis [1,5]. Unfortunately, as our patient presented with splenic rupture and due to the lack of contrast used on imaging, these findings were not clearly elucidated on her CT. Patients with acute splenic rupture may exhibit hyperattenuation on non-contrast CT, which was consistent with our patient’s radiologic findings [5]. Primary splenic angiosarcoma may be difficult to characterize on imaging alone, as CT findings are also consistent with other benign tumors, metastasis, lymphomas, or other sarcomas [5]. Ultimately, the diagnosis of splenic angiosarcoma is confirmed via exploratory laparotomy [1]. Due to hemodynamic instability, our patient underwent an emergency exploratory laparotomy to confirm the source of bleeding and subsequently remove the spleen.

Concluding that splenic rupture definitively worsens prognosis has been controversial; however, as this cancer has such a poor prognosis in general, there has not been sufficient evidence that splenic rupture significantly worsens a patient’s course. Immediately after splenectomy, our patient presented to the ED with suspected gastrointestinal bleed, which has been determined to be a very rare complication in patients with angiosarcoma, especially in the setting of metastatic disease [9]. The workup of our patient’s suspected gastrointestinal bleed resolved immediately upon hospital admission and was ultimately inconclusive, with evidence of metastasis only discovered months later.

Due to the aggressive nature of this cancer, the prognosis is relatively poor and generally fatal [1-3]. As we encountered with our patient, systemic therapy was not pursued due to the extent of disease and lack of increased survival rates. There is no specific treatment management plan for patients with splenic angiosarcoma; however, empiric trials of multi-agent chemotherapy have been offered to patients [1]. Our patient was considered for radiation therapy to the splenic bed, and this was subsequently deemed too toxic without true benefit to the patient. Those who are fortunate enough to have splenectomy prior to rupture or metastatic spread tend to obtain a higher survival rate [3]. Unfortunately, this was not the case for our patient, who was diagnosed secondary to splenic rupture. Many clinical trials are ongoing to find more favorable regimens for patients. Drugs targeting specific vascular pathways have shown clinical benefit [6]. Additionally, local/regional recombinant interleukin 2 (rIL-2) immunotherapy has shown to be beneficial in the limited studies performed, where T cells are activated to help the body overcome the disease and lymphokine-activated killer cells can better target rapidly proliferating endothelial cells [10].

Our patient was a poor candidate for therapy and, therefore, was closely monitored for recurrence or metastasis. She ultimately developed metastatic lesions to the liver, the most common site of metastasis in angiosarcoma. Following liver metastases, the lung, lymph nodes, and bone represent the next most common sites of spread, respectively [4]. Our patient had metastatic-proven disease within a year of initial diagnosis. Patients with splenic angiosarcoma have a 69%-100% metastasis rate [11]. Ultimately, this particular cancer has a dismal prognosis. A 14-year retrospective review of cases reported a five-year survival of 31% in their angiosarcoma patient study cohort [4]. Due to comorbidities and declining functional status, our patient pursued hospice.

## Conclusions

Primary splenic angiosarcoma represents a rare malignancy that can present with a variety of non-specific complaints such as abdominal pain, dyspnea, and weakness. Splenomegaly or the presence of an abdominal mass are common physical exam findings. A rare complication of this disease is splenic rupture, which may yield a worsened prognosis. Some studies suggest that prophylactic splenectomy has benefits in survival; however, due to the vascular nature of cancer, regardless of surgery, the rate of metastatic spread remains high. The goal of this case report is to perform a literature review of this rare condition, as well as improve upon the diagnosis of splenic angiosarcoma by considering this malignancy as a differential in patients who present with splenic rupture. With additional knowledge of this disease and further development of clinical trials, this may lead to earlier diagnosis with prophylactic splenectomies, ultimately reducing the occurrence of deadly complications from the primary tumor in these patients.

## Appendices

Reference ranges

WBC: 3.5 - 10.3 x 10^3/μL

Hb: 11.8 - 15.8 g/dL

Hct: 34.6 - 46.2%

Platelets: 140 - 440 x 10^3/μL

INR: 0.9 - 1.10

BUN: 8 - 20 mg/dL

Creatinine: 0.6 - 1.2 mg/dL

Glucose: 70 - 110 mg/dL

Lactate: 0.5 - 2.0 mmol/L

AST: ≤ 35 U/L

ALT: ≤ 52 U/L

ALP: 20 - 130 U/L
